# Adolescents’ smoking experiences, family structure, parental smoking and socio-economic status in Ciudad Juárez, Mexico

**DOI:** 10.1186/s12939-016-0323-y

**Published:** 2016-02-20

**Authors:** Yelena Bird, Hugo Staines-Orozco, John Moraros

**Affiliations:** School of Public Health, University of Saskatchewan, 104 Clinic Place, E-Wing Health Sciences, Room 3322, Saskatoon, SK S7N 5E5 Canada; Division of Biomedical Sciences, Universidad Autónoma de Ciudad Juárez, Juárez, Mexico

**Keywords:** Adolescents, Smoking, Secondhand smoke exposure, Family structure, Parental smoking, Socioeconomic status, Mexico

## Abstract

**Background:**

Cigarette smoking is the leading cause of preventable death worldwide. Tobacco use and secondhand-tobacco smoke (SHS) exposure are classified as a pediatric disease. In Mexico, the prevalence of smoking has decreased among adults but paradoxically increased among adolescents, particularly among young females. This study was designed to determine the association between adolescents’ smoking experiences (smoking behaviors and second hand smoke [SHS] exposure), family structure, parental smoking and socio-economic status (SES) in Ciudad Juárez, Mexico.

**Methods:**

This is a cross-sectional, population-based study. Data was collected from sixth-grade students (*N* = 506) attending school in Ciudad Juárez, Mexico. Descriptive analyses were conducted. The relationship between key outcome variables (adolescents smoking and SHS exposure) and independent variables (family structure, parental smoking, and SES level) were examined. Adjusted odds ratios were calculated. Multiple logistic regression analysis was performed while controlling for possible confounders (i.e. gender and age).

**Results:**

The overall prevalence of ever/lifetime smoking and SHS exposure at home was 29.6 and 41.1 %, respectively. Results of the logistic regression analysis show that being a member of a non-intact family [(OR = 2.20; 95 % CI = 1.21–3.90) and (OR = 2.45; 95 % CI = 1.19-4.10) respectively], having parents who smoke [(OR = 4.41; 95 % CI = 2.15–5.46) and (OR = 4.95; 95 % CI = 2.25-7.12) respectively], and living in low SES setting [(OR = 1.73; 95 % CI = 1.43–3.30) and (OR = 1.99; 95 % CI = 1.16-4.00) respectively] are significantly associated with ever smoking and SHS exposure at home among sixth grade students.

**Conclusions:**

The findings of our study show that tobacco use and SHS exposure are strongly associated with adolescents living in low SES, non-intact households that have parents that smoke. To be effective, tobacco strategies specifically tailored for this particularly vulnerable group of adolescents would require a comprehensive, multi-faceted approach centered on prevention, cessation and protection.

## Background

Cigarette smoking is the leading cause of preventable death worldwide [1]. Tobacco use and secondhand-tobacco smoke (SHS) exposure are classified as a pediatric disease [2]. Globally, it is estimated that nearly 100,000 adolescents begin smoking every day with the vast majority (approximately 80 %) of them from low-income countries [3]. If current trends continue, more than 250 million adolescents alive today will die prematurely from tobacco-related diseases [4, 5].

In Mexico, the prevalence of smoking has decreased among adults but paradoxically increased among adolescents, particularly among young females [6, 7]. It is reported that almost one million adolescents use tobacco daily in Mexico. [7] Most first time use of tobacco occurs in adolescence and because nicotine is addictive, adolescents who smoke regularly are likely to become lifelong adult smokers [8]. These are troubling trends with significant consequences on the economy and healthcare system of Mexico. On average, a smoker in Mexico would have to spend nearly 5 % of their income (national median) to purchase 10 of the cheapest cigarettes each day [6]. On a national level, approximately 50,000 people die prematurely due to tobacco related diseases [9] and the direct medical expenses attributable to tobacco use are estimated to cost Mexico $5.7 billion (USD) annually [6].

Tobacco use and SHS exposure have immediate and long term effects on the health of adolescents. The immediate effects include increases in respiratory symptoms and reductions in lung function [10, 11]. It has been reported that respiratory problems such as asthma, cough, phlegm, and wheezing are significantly more likely to occur among adolescent smokers and those exposed to SHS than their non-smoking counterparts [10]. Similarly, active smoking and SHS exposure in adolescence can have a significant effect on several pulmonary function parameters, including reductions in FVC, FEV_1_, and FEF_25-75%_ [11]. Additionally, early age of smoking initiation is known to increase the lifetime risk of developing a variety of cancers [12], cardiovascular diseases [13], and respiratory illnesses especially among women [14].

Tobacco use in adolescence is the result of a set of complex interactions between youth and their social environment. While several theories have been examined in an attempt to identify the factors that influence smoking behavior in adolescents, no consensus has been reached. Several research studies have indicated that low socio-economic status, single parent homes and/or the presence of one or both parent(s) being smokers exert significant influences on the acquisition of tobacco use habits by adolsecents [15–18]. However, the role that family structure and socio-economic status play on the smoking behaviors of Mexican adolescents has not been sufficiently studied.

Public health efforts to reduce and prevent tobacco use among adolescents in Mexico require a comprehensive approach and multi-faceted strategy that address not only the physical dependence but also the social context of the behavior. Therefore, it is critically important to identify and understand the degree to which familial and socio-economic conditions influence the development of smoking habits among adolescents. The present study was designed to determine the association between adolescents’ smoking experiences (smoking behaviors and SHS exposure), family structure, parental smoking and socio-economic status in Ciudad Juárez, Mexico.

## Methods

### Study setting

The present study was conducted in Ciudad Juárez, Mexico. Ciudad Juárez is one of the major cities on the US-Mexican border and the fourth largest city in Mexico, with an estimated population of 1.5 million people in 2010 [19]. Mexico has a relatively young and growing population of approximately 122 million people with nearly 10 % between the ages of 12 and 16 years old [20]. This age group has been identified by the tobacco industry as a high priority marketing/consumer target [21].

Some of the factors that contribute to making adolescents living in Mexico a particularly vulnerable risk group for tobacco use include but may not be limited to easy access to cigarettes [22], peer pressure [23], social acceptability [24], parental use [24, 25], aggressive tobacco industry marketing [21, 26], and most importantly, the fact that even though smoking in enclosed public places (i.e., offices, schools, government buildings, and restaurants) is strictly prohibited, compliance is low [27–29]. Further exacerbating the problem is the reality that in Mexico, tobacco companies are allowed to sell “kiddie packs” and even “individual cigarettes” mainly by street vendors (many of them kids themselves) [28, 29]. A recent study reported that single cigarettes in Mexico were widely accessible and growing in popularity and use among the general population including minors [30]. This makes regulation of tobacco sells and enforcement of anti-tobacco laws particularly difficult.

### Study design

This is a cross-sectional study based on a broader population study [31]. The present study was conducted to examine the association between the smoking experiences (behaviors and SHS exposure), family structure, parental smoking and socio-economic status among sixth-grade students (ages 11-13 years old) in Ciudad Juárez, Mexico. A list of middle schools within the city limits of Ciudad Juárez was obtained with the kind assistance of the Ministry of Education.

The methodology for the present study was adapted from the Global Youth Tobacco Survey (GYTS) [32]. Specifically, it uses a two-stage cluster sample design to produce representative samples of sixth grade students (ages 11–13 years old) attending middle school in Ciudad Juárez, Mexico. In the first stage, schools were selected randomly from a list provided by the Ministry of Education based on their proportional probability of sixth grade students enrolled in the specified setting (public or private) and SES (low, middle and high) category. In the second sampling stage, classes within each selected school were randomly selected. All students present on the day of the survey were eligible to participate.

In the present study, replicate weights were used to adjust for the varying probabilities of sample selection (student, class, and school settings). The full study sample was broken up into a series of subsamples by using the key outcomes of interest and the non-overlapping strata under consideration. Then estimates were calculated for the full sample and in each subsample to account for within cluster variance.

Socioeconomic economic status (SES) information was obtained from the Ministry of Education of Ciudad Juárez, Mexico. Based on this information, all middle schools were stratified by SES to low (<10,000 Mexican pesos, average annual household income), middle (10,000 - 25,000 Mexican pesos, average annual household income per year), and high (>25,000 Mexican pesos, average annual household income; $1US dollar =16.5 Mexican pesos in 2015) [31].

Permission was obtained from the corresponding educational authorities in Ciudad Juárez, Mexico to conduct the study. The questionnaire application was performed in the classroom and in the presence of the corresponding teacher. In addition, informed consent was obtained from all adolescents and their parents or guardians prior to their participation in the study. Students had the right to withdraw from the study at any point in time and without any penalties even after completing the questionnaire.

### Ethics statement

All study procedures and instruments were reviewed and approved by the Institutional Review Boards at New Mexico State University (USA) and Universidad Autónoma de Ciudad Juárez (Mexico). All sixth-grade students present on the day of administration of the survey (*N* = 506) were eligible to participate. No monetary or nonmonetary incentives were offered to the participating schools or students.

### Survey instrument

The instrument used in this study was an adaptation of the GYTS [32]. The survey was specifically developed for use with middle school students only. The GYTS was particularly suitable because it could be completed in class during a short period of time. The core questionnaire includes 54 questions covering eight broad topics or categories. The portion of the questionnaire used in this study consisted of four sections that pertained to the adolescents: a) sociodemographic characteristics, b) smoking experiences (behaviors and SHS exposure), c) family structure, and d) parental smoking. All the items were categorical, closed-format questions. The instrument has been explained extensively elsewhere [31].

### Data collection

Investigators handed out paper copies of the survey and scantron sheets to all students. To maintain anonymity and confidentiality, respondents were instructed not to place names, identification marks, or numbers anywhere on the instrument. Upon completion, the respondents deposited their surveys into a large, brown, unmarked envelope that was immediately sealed and removed from the classroom.

### Data analysis

All data analyses were conducted using SPSS and STATA statistical software packages. Frequencies, means, and standard deviations were used to describe the participants and their responses on the survey instrument. The key outcomes under investigation were smoking behaviors and SHS exposure among sixth grade students. The relationship between the key outcome variables and independent variables such as family structure, parental smoking, and SES level were examined. Adjusted odds ratios (OR) and their 95 % confidence intervals (CI) were calculated. Multiple logistic regression analysis was performed while controlling for possible confounders (i.e. gender and age). Differences in proportions were considered statistically significant at *p* < 0.05

## Results

### Study population characteristics

There were 506 sixth grade students who participated in the study. The response rate (i.e. completed and returned surveys) was 100 %. The study population characteristics are presented in Table 1. The majority of the students were 12 years old and nearly half were male. Smoking prevalence varied by age, with older students having a higher prevalence. Nearly a third of male students reported having ever smoked; the proportion was slightly lower among females. More than 30 % of the students attending public school had ever smoked; the proportion was lower for those attending private schools. Students living with parents who smoke in a non-intact, low SES household had the highest smoking prevalence.

**Table 1:** Sociodemographic Characteristics among Adolescents

Sociodemographic Characteristics	N (506)	Percent %	Smoking Prevalence^a^
Gender			
Boys	242	47.8	32.6
Girls	264	52.2	26.9
Age			
11 Years Old	20	3.9	15.0
12 Years Old	471	93.1	29.7
13 Years Old	15	3.0	46.6
School Setting			
Public	279	55.1	32.9
Private	227	44.9	25.6
Family Structure			
Non-intact	121	23.9	46.3
Intact	385	76.1	24.4
Parental Smoking			
Yes	195	38.5	54.0
Father only	96	18.9	
Mother only	54	10.7	
Both parents	45	8.9	
No	311	61.5	14.5
Socioeconomic Status			
Low (<10,000 Mexican pesos)^b^	177	35.0	37.3
Middle (10,000-25,000 Mexican pesos)^b^	151	29.8	27.2
High (>25,000 Mexican pesos)^b^	178	35.2	24.2

^a^Smoking prevalence was defined as a student ever having smoked a full cigarette

^b^$1US dollar =16.5 Mexican pesos in 2015

### Smoking behaviors

Two questions were used to determine the smoking behaviors of the student participants. Based on the definition used in the National Addiction Survey Mexico 2002 [33], we considered adolescents as current (i.e. active) smokers when they reported themselves to be smokers at the time of completing the survey. The first question sought to determine the number of current smokers among the sixth grade students. Of the 506 participants, 26.1 % [*N* = 132] reported being current smokers. Of the current smokers 54 % [*N* = 71] were boys and 46 % [61] were girls. The second question assessed how many students had ever smoked a full cigarette in their life time and was used to determine the smoking prevalence in our study. Of all the students surveyed, 29.6 % [*N* = 150] indicated they had smoked a full cigarette. Of the students who had engaged in this behavior, 53 % [*N* = 79] were boys and 47 % [*N* = 71] were girls. Finally, it is worthy to note that 55.3 % [*N* = 83] of the smokers indicated they had initiated smoking at or before the age of 10 years old (Table 2).

**Table 2:** Smoking Behaviors and SHS Exposure among Adolescents by Family Structure, Parental Smoking, and Socioeconomic Status

	Family structure^a^		Parental smoking^a^		SES^a^		
	Non-Intact	Intact	Yes	No	Low	Middle	High
	(*N* = 121)	(*N* = 385)	(*N* = 195)	(*N* = 311)	(*N* = 177)	(*N* = 151)	(*N* = 178)
Smoking behaviors							
- Ever smoked?	46.3^b^	24.4	54.0^b^	14.5	37.3^b^	27.2	24.2
- Currently smoke?	37.2^b^	22.6	47.7^b^	12.5	35.0^b^	23.2	19.7
SHS exposure							
**-** Do you live in a home where in the last 7 days others smoke in your presence?	56.2^b^	36.4	73.3^b^	20.9	56.5^b^	39.1	27.5
- In the last 7 days were you around others who smoked in your presence in places outside your home?	71.1^c^	47.5	82.6^b^	34.7	65.0^c^	46.4	47.2

^a^ Percent of students answering questions in the affirmative (“yes”)

^b^ Correlation is significant at *p* < 0.01

^c^ Correlation is significant at *p* < 0.05

### Secondhand smoke exposure

Two questions were used to determine the SHS exposure of the student participants. We considered adolescents to be exposed to SHS if they answered affirmative one of two questions. The first question asked the sixth grade students whether they live in a home where in the last 7 days others smoke in their presence. Of the 506 participants, 41.1 % [*N* = 208] reported being exposed to SHS at home. The second question asked the sixth grade students whether in the last 7 days they were around others who smoked in their presence in places outside the home. Of all the students surveyed, 53.2 % [*N* = 269] indicated they had been exposed to SHS outside their home with most of them being boys (Table 2).

### Family structure

This variable was defined based on the presence or absence of the biological father, the biological mother, or both biological parents in the adolescent’s home. This variable was coded as 1 = non-intact family (absence in the home of one or both biological parents) and 0 = intact family (presence in the home of both biological parents). Of the 506 participants, 76 % [*N* = 385] reported living in intact homes. Of those who lived in non-intact homes [*N* = 121], 86 % lived with their mothers (Table 2).

### Parental Smoking

This variable was determined on the basis of the response provided by the participating sixth grade students to the following question: Do your parents (father, mother or both) smoke? This independent variable was coded as 1 = parents who smoke (presence in the home of either a father or mother who smoked) and 0 = non-smoker parents (neither of the parents smoked). Of the 506 participants, 31 % [*N* = 157] reported living in homes with who smoke. Of those, 63 % [*N* = 99] reported their fathers being smokers, 12 % [*N* = 19] reported their mothers being smokers and the remaining 25 % [*N* = 39] reported having both parents as smokers (Table 2).

### Multiple Regression Analysis

We examined the associations between the outcome measures (i.e., smoking behaviors and SHS exposure) and key characteristics of the participant students (i.e., family structure, parental smoking, and SES) by using logistic regression models, as shown in Tables 3 and 4. The outcome measures are presented individually and the key characteristics were treated as independent variables in our models. Odds ratios are shown in relation to a reference category for each variable.

**Table 3:** Effect of Family Structure, Parental Smoking, and Socioeconomic Status on Smoking Behaviors among Adolescents

	Smoking behaviors					
	Ever smoked?			Currently smoke?		
Independent Variable	OR^a^	95 % CI	*P*-value	OR^a^	95 % CI	*P*-value
Family Structure						
Non-intact	2.20	1.21-3.90	0.01	2.00	1.41-4.45	0.01
Intact^b^						
Parental Smoking						
Yes	4.41	2.15-5.46	0.01	4.10	2.05-6.16	0.01
No^b^						
Socioeconomic Status						
Low (<10,000 Mexican pesos)	1.73	1.43-3.30	0.01	1.81	1.27-3.42	0.01
High^b^ (>25,000 Mexican pesos)						

*95 % CI* 95 % confidence interval; *OR* odds ratio

^a^Adjusted for gender and age

^b^Reference category

**Table 4:** Effect of Family Structure, Parental Smoking, and Socioeconomic Status on SHS Exposure among Adolescents

	SHS Exposure					
	Do you live in a home where in the last 7 days others smoke in your presence?			In the last 7 days were you around others who smoked in your presence in places outside your home?		
Independent Variable	OR^a^	95 % CI	*P*-value	OR^a^	95 % CI	*P*-value
Family Structure						
Non-intact	2.45	1.19-4.10	0.01	1.88	1.24-3.80	0.05
Intact^b^						
Parental Smoking						
Yes	4.95	2.25-7.12	0.01	3.91	2.05-5.96	0.01
No^b^						
Socioeconomic Status						
Low (<10,000 Mexican pesos)	1.99	1.16-4.00	0.01	1.58	1.12-3.84	0.05
High^b^ (>25,000 Mexican pesos)						

*95 % CI* 95 % confidence interval; *OR* odds ratio

^a^Adjusted for gender and age

^b^Reference category

Results of the logistic regression analysis show that being a member of a non-intact family [(OR = 2.20; 95 % CI = 1.21–3.90) and (OR = 2.45; 95 % CI = 1.19-4.10) respectively], having parents who smoke [(OR = 4.41; 95 % CI = 2.15–5.46) and (OR = 4.95; 95 % CI = 2.25-7.12) respectively], and living in low SES setting [(OR = 1.73; 95 % CI = 1.43–3.30) and (OR = 1.99; 95 % CI = 1.16-4.00) respectively] are significantly associated with ever smoking and SHS exposure at home among sixth grade students.

## Discussion

The present study demonstrates that sixth grade students living in low SES, non-intact family households and who have parents that smoke are significantly more likely to be smokers and be exposed to SHS in Ciudad Juárez, Mexico.

According to the findings of our research, adolescents residing in a low SES setting were 2.7 times more likely to have ever smoked and 1.9 times more likely to be current smokers when compared with those residing in a high SES setting. This is consistent with the most recent findings by Kuipers et al. [34], and the original findings from Conrad et al. [35], that showed a strong inverse association between SES and adolescent smoking in 76 % of 21 prospective studies reviewed. It is interesting to note that of the 150 students who indicated they had tried smoking in our study, 88 % reported being active smokers and 55 % initiated smoking at or before the age of 10 years old. By starting to use tobacco at such a young age, it makes adolescents particularly vulnerable on several fronts.

Tobacco’s highly addictive properties make it more likely that a number of experimenting adolescents may become life-long, adult cigarette users [8]. It has been reported that most people who become regular smokers initiate and establish their smoking habits during adolescence [36]. In addition to the well documented health concerns [10–14], buying tobacco causes financial hardship on low SES adolescents as it robs them and their families of the possible resources they need to rise out of poverty [7]. It is estimated that the poorest 20 % of households in Mexico spend nearly 11 % of their income on tobacco. [37]

Census data over the last decade have shown that the number of non-intact (i.e. single-parent) families (predominantly led by mothers) has risen in Mexico. [38]. This social phenomenon has been associated with the development of increased risk behaviors among affected adolescents [15, 39–41]. Similarly, our study shows that adolescents who live in non-intact family households were 2.2 times more likely to have ever smoked and 2.0 times more likely to be current smokers when compared with those residing in intact households (i.e. living with both biological parents). This effect may be due to a number of socio-cultural factors.

In Mexico, tobacco use is generally viewed as a socially acceptable behavior for boys but not so for girls. These views may help promote smoking among adolescents from both genders but for potentially different sociocultural reasons. For many young boys and girls alike, especially those living in low SES, non-intact family households, smoking may represent a rite of passage from childhood to adulthood [42]. For boys, smoking plays into and reinforces the sociocultural concept of “machismo.” Tobacco is used in these instances by boys as a way of asserting their masculinity and proving their power and fearlessness [43]. For girls, it may be a reflection of trying to actively rebel against the sociocultural concept of “marianismo.” Traditional Latin American societies such as the one seen in Mexico tend to think that 'good girls' do not smoke [44]. Therefore young girls may deliberately smoke to show that they will not be subjugated to the rules of the past. For many of them smoking signals entry into womanhood by giving them the appearance of being stylish, sexy and independent [45, 46].

There are a number of other factors that can provide appreciable meaning and context of smoking among Mexican adolescents. Social smoking among adolescents provides opportunities for bonding and group membership that may transition from one time or space (e.g., day time in schools) to another (e.g., evening and nights during parties and clubbing) [47]. Even though most adolescents minimize the importance of peer influence over their smoking behavior, they readily describe social interchange networks, in which simultaneous cigarette smoking and sharing occurs [23]. These findings provide evidence for the operation of self-selection and peer-influence processes on adolescent smoking behaviors [48, 49]. Many adolescents also reported smoking cigarettes to escape boredom [50], relieve stress [51], and for sensation seeking purposes [52]. These connotations are strongly reinforced among adolescents by the tobacco industry through targeted advertising and mass media campaigns [21, 26, 53].

Additionally, living in a non-intact home (mainly with their mother) may lead the adolescent to have a lower household income (i.e. low SES), experience less or even lax supervision by the single parent and socially rely more heavily on peers. Conceivably, the single parent may be more tolerant of the behavior or even too busy working to be fully aware of their adolescent’s activities outside the home [41, 54, 55]. Adolescents from non-intact homes may be particularly susceptible to peer social influences given the increased importance of school and peer groups in their lives [56, 57]. All these factors and many others, conspire to create a vicious cycle for many adolescents for whom their family structure (i.e. non-intact households) influences their socio-economic status (i.e. low) and makes them particularly vulnerable to tobacco initiation and long term use.

Based on the results of our study, adolescents who live in a household with parents that smoke were 4.4 times more likely to have ever smoked themselves and 4.1 times more likely to be current smokers when compared with those residing in non-smoking households. A number of studies have provided support for this association [18, 58, 59]. Parents serve as role models for many adolescent behaviors including smoking. The low SES environment puts parents (especially the ones leading non-intact households) at risk to be smokers themselves and by way of modeling and mimicry adolescents may become influenced to copy the behavior. The presence of parents that smoke in the household may be perceived by adolescents as an indication that such behavior is acceptable and a sign that smoking is not that harmful to one’s health [60].

Even for adolescents who do not smoke, their potential exposure to SHS in their homes poses a serious risk to their health. In the present study, SHS exposure for our adolescent participants was 41.1 % at home and 53.2 % outside of it. The resulting tobacco smoke exposure increases their risk to suffer from acute respiratory infections, otitis media, decreased lung function, exacerbation of asthma, hospitalizations, dental caries, mental health problems, cognitive deficits, and even school absenteeism and poorer academic performance [61–66].

In our study, the prevalence of parental smoking was quite high (31 %). Based on the results of our research, adolescents whose parents smoke were 4.9 times more likely to have been exposed to SHS in their own home and 3.9 times more likely outside of it, when compared with those residing in non-smoking households. Our findings lend support to those reported in a systematic review of the literature, which shows parental smoking and low SES to be independently and significantly associated with children’s SHS exposure in their home [11].

In Mexico, smoke-free housing policies are not common and public smoking bans are not regularly enforced [67]. This indicates that public health and education professionals have an opportunity to play an important role in reducing SHS exposure among adolescents with counseling strategies centered on parents that emphasize increased awareness, education about smoke-free housing policies and public smoking bans, and smoking reduction and cessation efforts.

### Limitations

This study has a number of limitations. The students who participated in the study may have differed in important ways from those who did not participate. Even though our participation rate was 100 % on the days of the survey, there probably were a number of students who were absent from school on those days. Additionally and more importantly, there are growing numbers of middle school age children in Mexico (close to one-in-three by some reports), who may attend school irregularly or not at all [38]. Despite the fact we did not collect data from non-participant adolescents because they were either absent or do not attend school, the literature suggests their smoking rates are even higher than those who attend school [68]. The study used a purposive sampling method of sixth grade students from selected middle schools. This in turn may have compromised our ability to generalize the results of our study to other grades and middle schools. Nevertheless, our sampling scheme ensured that data were collected from representative public and private middle schools located in low, middle and high SES neighborhoods in Ciudad Juárez, Mexico. Finally, smoking was not determined by an objective measurement, such as biochemical validation, but rather on the basis of self-reported behaviors by the adolescent participating students. However, research has shown the validity of self-reported smoking to be consistently high and thus, biochemical measurements for validation purposes may not be justified [69].

## Conclusions

In Mexico, tobacco use has significant implications for the nation’s economy and public health. This holds especially true among its adolescent population. However, adolescents face an uphill battle in their fight against tobacco. Unlike many other health conditions, the severe effects of tobacco use do not clinically manifest themselves until several decades later. If one couples this reality with the adolescents’ perceived sense of health invincibility, it is understandable why many adolescents do not consider tobacco to pose a serious threat to their health [8].

Our study findings show that tobacco use and SHS smoke exposure are strongly associated with adolescents living in low SES, non-intact households that have parents that smoke. To be effective, tobacco strategies specifically tailored for this particularly vulnerable group of adolescents would require a comprehensive, multi-faceted approach centered on: a) prevention – helping to stave off adolescents from starting to use tobacco, b) cessation – helping the active adolescent smokers (and their parents) to quit, and c) protection – safeguarding adolescents from the harmful effects of secondhand smoke exposure by strengthening policies and enforcing regulations.
